# OA12 Recurrent knee swelling- all is not crystal clear

**DOI:** 10.1093/rap/rkae117.012

**Published:** 2024-11-05

**Authors:** Koushan Kouranloo, Yik Long Man, Louise C Pollard

**Affiliations:** Department of Rheumatology, University Hospital Lewisham, London, United Kingdom; School of Medicine, University of Liverpool, Liverpool, United Kingdom; Department of Rheumatology, University Hospital Lewisham, London, United Kingdom; Department of Rheumatology, University Hospital Lewisham, London, United Kingdom

## Abstract

**Introduction:**

Septic arthritis can lead to permanent joint destruction, with a mortality rate of 7-15% despite antibiotic treatment. However, it can be challenging to diagnose, as synovial fluid cultures are not always positive. In recent years, 16S Polymerase Chain Reaction (PCR) has been more accessible, which can be helpful when there is diagnostic uncertainty.

We present a case of knee swelling in an elderly man with known haemochromatosis. Although initial synovial fluid (SF) analysis showed crystals, his swelling reaccumulated despite treatment for Calcium Pyrophosphate Disease (CPPD). There was ongoing clinical concern of septic arthritis. 16S-PCR identified *Neisseria gonorrhoea*, confirming gonococcal arthritis.

**Case description:**

A 77-year-old Caucasian man, with a background of haemochromatosis, presented with acute right knee and left wrist swelling. He had no fevers, rashes or genitourinary symptoms. He had a regular male partner.

Initial blood tests showed a CRP of 119 mg/l and white cell count of 12.8 x10^9^/l. Knee X-ray demonstrated chondrocalcinosis. Knee arthrocentesis showed turbid fluid. He was treated for presumed CPPD with Colchicine and covered with intravenous (IV) Flucloxacillin whilst awaiting SF results, which demonstrated numerous pus cells, calcium pyrophosphate crystals and a negative Gram-stain.

After two days, the left wrist improved, but his right knee swelling reaccumulated. He remained apyrexial, and the blood and SF cultures were negative. However, his inflammatory markers continued to rise (CRP 192 mg/l), increasing concerns about septic arthritis. A Sexually Transmitted Infection (STI) screen was requested. He was referred to Orthopaedics, who performed a second knee arthrocentesis. The SF once again showed no infection; Orthopaedics felt that septic arthritis was unlikely, and a washout was not performed. As such, Prednisolone 20 mg once daily was commenced for presumed inflammatory arthritis. Despite this, his knee swelling recurred, his CRP remained elevated at 165 mg/l and the knee was again aspirated. The clinical suspicion of septic arthritis remained high, and a 16S-PCR was requested.

His throat swab subsequently returned positive for *Neisseria gonorrhoea*, and he was given IV Ceftriaxone, and his CRP improved to 49 mg/l. His 16S-PCR returned negative, suggesting that he had reactive arthritis secondary to a gonococcal infection. He was followed up in the Rheumatology clinic and made a full recovery with no residual joint problems.

To our surprise, the Microbiology team resent the initial SF sample specifically for *Neisseria gonorrhoea* 16S-PCR, when in receipt of the positive STI screen. This was positive, and ultimately, this man had gonococcal arthritis despite the multiple negative SF cultures.

**Discussion:**

Disseminated gonococcal infections remain rare, accounting for just 0.5-3% of *Neisseria gonorrhoea* cases. Gonococcal arthritis is the most frequent of the systemic manifestations, accounting for 30-90%, depending on the population studied. Clinical features can also comprise tenosynovitis, skin lesions and constitutional symptoms. As with all cases of septic arthritis, it is essential to promptly detect and treat it to prevent joint destruction and death. In the UK, gonorrhoea infections are at their highest since records began. It is therefore important for Rheumatologists to be vigilant about gonococcal arthritis.

This case was diagnostically challenging. The most common cause of acute wrist and knee swelling in an elderly inpatient is crystal arthritis. Furthermore, he had an established diagnosis of haemochromatosis which is associated with CPPD. This was confirmed on the knee X-ray and SF analysis. However, CPPD is usually self-limiting, so it was concerning that his knee swelling reaccumulated, and his CRP continued to rise, despite treatment with Colchicine.

The diagnosis of CPPD was therefore revisited; an Orthopaedic opinion concerning septic arthritis was requested, as well as an STI screen and 16S-PCR. IV Flucloxacillin was never discontinued due to the ongoing clinical concern of septic arthritis, despite the negative blood and SF cultures. His knee swelling did not improve with Prednisolone, and the clinical picture only improved when he was treated with Ceftriaxone, after his throat swab confirmed *Neisseria gonorrhoea* infection.

While 16S-PCR remains a highly sensitive and specific method to detect bacteria, its accuracy is not 100% and generally lower than targeted real-time PCR assays. In this case, *Neisseria gonorrhoea* 16S-PCR was positive when specifically requested. This would explain why his knee swelling only improved when he was eventually given Ceftriaxone.

**Key learning points:**

• Septic arthritis is a clinical diagnosis and synovial fluid cultures can be negative in 25-50% of cases, even if taken before the initiation of antibiotics. There may be no fever and the inflammatory markers may not always be markedly elevated. Where there is ongoing clinical concern of septic arthritis, 16S-PCR can be requested on SF samples, although the results currently take 1-2 weeks.

• Although 16S-PCR has a high sensitivity and specificity, their accuracy remains lower than targeted real-time PCR assays. In this case, *Neisseria gonorrhoea* 16S-PCR had to be specifically requested to clinch the diagnosis.

• It is always important to revisit the initial diagnosis when there is a lack of clinical improvement with treatment. Continuation of IV antibiotics when there is ongoing clinical concern about septic arthritis remains vital. In this case, the patient’s history was key, which prompted early STI testing.

• It is essential not to shy away from asking about genitourinary symptoms where indicated, and ascertaining a thorough sexual history. Multidisciplinary involvement, including Rheumatology, Orthopaedics, Genitourinary medicine and Microbiology is highly encouraged to ensure appropriate sampling and optimum treatment. This will ultimately result in reduced symptom burden and length of inpatient stay.

• Given the increased incidence of gonococcal infections, physicians should be aware of the presentation of, and how to diagnose this infection. Our case underlies the importance of thinking outside the diagnostic box and serves as a reminder to talk about traditionally “taboo” subjects.

